# Niemann-Pick Disease Type C Presenting as a Developmental Coordination Disorder with Bullying by Peers in a School-Age Child

**DOI:** 10.1155/2015/807591

**Published:** 2015-12-16

**Authors:** Ryo Suzuki, Atsushi Tanaka, Toshiharu Matsui, Tetsuki Gunji, Jun Tohyama, Aya Nairita, Eiji Nanba, Kousaku Ohno

**Affiliations:** ^1^Department of Pediatrics, Nagaoka Chuo General Hospital, 2041 Kawasaki-cho, Nagaoka, Niigata 940-8653, Japan; ^2^Department of Child Neurology, Nishi-Niigata Chuo National Hospital, 1-14-1 Masago, Nishi-ku, Niigata, Niigata 950-2085, Japan; ^3^Division of Child Neurology, Institute of Neurological Sciences, Faculty of Medicine, Tottori University, 36-1 Nishimachi, Yonago, Tottori 683-8504, Japan; ^4^Division of Functional Genomics, Research Center for Bioscience and Technology, Tottori University, 86 Nishimachi, Yonago, Tottori 683-8503, Japan; ^5^Sanin Rosai Hospital, 1-8-1 Kaike Shinden, Yonago, Tottori 683-8605, Japan

## Abstract

Niemann-Pick disease type C (NPC) is a rare progressive neurodegenerative disorder, often with onset after normal early childhood development. Juvenile onset NPC patients slowly develop cerebellar symptoms and cognitive impairment and often experience difficulties at school. However, these problems may be overlooked due to the unpublicized nature of NPC, given that it is a rare metabolic disorder. In this report, we present an 11-year-old male NPC patient, who suffered from clumsiness and difficulties in attention and academic and social skills. His symptoms were initially considered to be due to developmental coordination disorder (DCD) coexisting with bullying by peers. DCD is a type of neurodevelopmental disorder defined according to DSM-IV and is characterized by clumsiness that interferes with academic achievement and social integration not due to other general medical conditions. However, a detailed investigation of the patient suggested that the problems could be attributed to the onset of NPC. Clinicians should keep neurodegenerative disorders as differential diagnosis of children with multiple school problems.

## 1. Introduction

Niemann-Pick disease type C (NPC) is a lysosomal lipid storage disorder with an accumulation of sphingomyelin in reticuloendothelial and parenchymal tissues [1]. The disease is inherited as an autosomal recessive trait and two separate disease gene loci have been identified [2, 3]: the* NPC1* gene on chromosome 18q11-q12 and the* NPC2* gene on chromosome 14q24.3. Clinical features of NPC include a wide spectrum of visceral and neurological signs and symptoms, with age at onset ranging from the perinatal period to late adulthood [1]. For many NPC patients, the onset of the disease is during the juvenile period after normal early development, and they commonly have problems in school, such as poor writing and impaired attention [1]. Here, we report the case of a Japanese male patient who was originally thought to have developmental coordination disorder (DCD), which is characterized by clumsiness that interferes with academic achievement and social integration not due to other general medical conditions [4], coexisting with bullying by peers. However, neurological examination showed typical features of a juvenile onset form of NPC. This case suggests that the early phases of neurodegenerative disorders with multiple school problems can be overlooked or misdiagnosed because of a low index of suspicion among clinicians. Clinicians should consider NPC as a possible cause of degenerative disorders when seeing children with progressive multiple school problems.

## 2. Case Report

**Figure 1:**
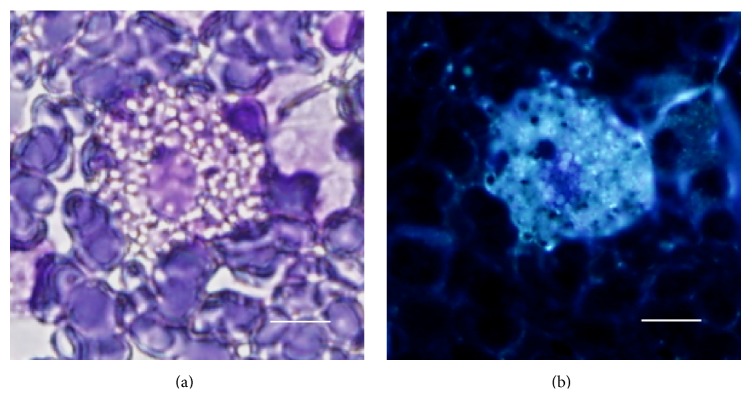
(a) High power view of the May-Giemsa-stained bone marrow aspirate. A large macrophage laden with sphingomyelin gives the cytoplasm a foamy appearance. (b) Positive filipin staining of a foam cell is observed on the same field as (a) (scale bar: 10 *μ*m).

**Figure 2:**
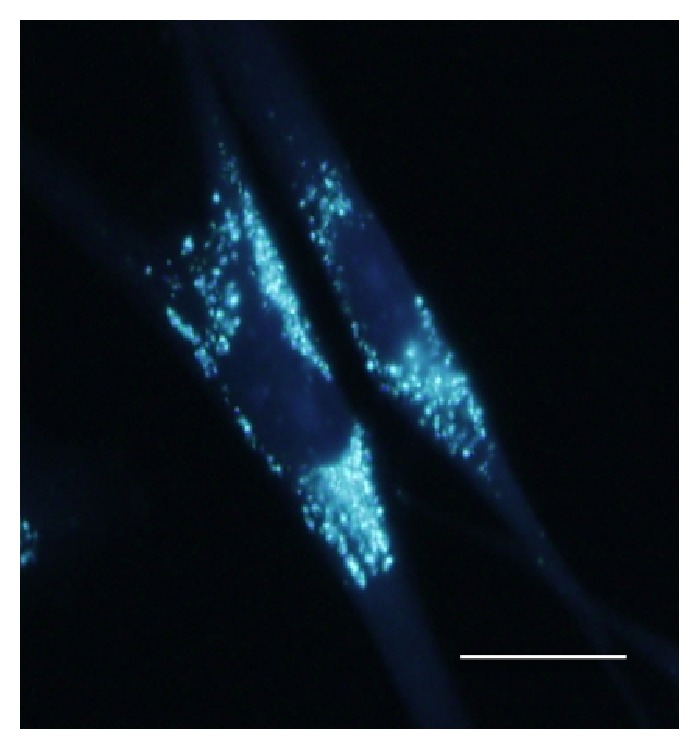
Filipin staining is positive in the patient's cultured skin fibroblast cells (scale bar: 20 *μ*m).

A Japanese boy without a familial history of NPC or consanguineous marriages was born at a weight of 3040 g by vacuum extraction after 40-week gestation. He had been admitted to a newborn nursery for 3 weeks because of meconium aspiration syndrome and prolonged neonatal jaundice. Thereafter, he grew up with normal gross motor milestones: head control attained at 4 months, sitting without support at 7 months, crawling at 8 months, and walking independently at 15 months. After entering elementary school, he had no problem with his schoolwork and initially built successful relationships with his friends, but then he began to be bullied at school when he was around 9 years old. He suffered from pushing, hitting, verbal taunts, and social exclusion by peers. Afterwards, he began to show a depressed appearance and less positive social interaction with classmates and spent more time on his own than with peers. Furthermore, he started to gradually develop difficulties in sustaining close attention to details, declining grades, and clumsiness that interfered with handwriting and activities such as riding a bicycle and playing catch. At 10 years of age, he presented to our pediatric outpatient department complaining of the problems above; however, we misdiagnosed him at this time as having DCD with bullying by peers. Then, we suggested a “wait-and-see” approach after trying supportive care involving the family, school, and other individuals in the child's environment. One and a half years later, at the age of 11 years, he presented to us again because of his progressive clumsiness and notably deteriorated school performance with ongoing bullying episodes. Upon examination, no significant signs were found except for hepatomegaly. On neurological examination, he was alert and oriented, although his speech was slurred with impaired pronunciation. He showed mild facial dyskinesia, slight dysarthria and dysphasia, and exaggeration of deep tendon reflexes in both extremities. Finger-to-nose test was performed correctly, but his movements were slow and slightly awkward. Fine and gross motor clumsiness had a negative impact on his skills in writing, running, and riding a bike. Although guided and voluntary eye movements were normal, he was unable to perform downward saccadic eye movements in an ophthalmic evaluation, which is a well-known initial sign of NPC [1]. His full-scale IQ was 63 on the Wechsler Intelligence Scale for Children-III. Routine laboratory tests showed normal results, including blood and cerebrospinal fluid, plasma amino acid analysis, and urine organic acid analysis. Cerebral magnetic resonance imaging showed no abnormality. Suspecting NPC from his progressive symptoms, we performed a bone marrow examination as a preliminary test [5]. Cytology of his bone marrow aspirate showed foam cells (Figure 1), supporting the diagnosis of NPC. Furthermore, cytochemical studies on cultured skin fibroblasts showed a significant accumulation of unesterified cholesterol in perinuclear vesicles by cytochemical staining with filipin (Figure 2). In addition, a molecular study of the NPC gene was carried out. Sequencing of all 25 exons of* NPC1* and their boundaries was performed on genomic DNA from the patient. We found the compound heterozygous mutations p.P836fsX838 [c.2506 (or 2507) del C] at exon 16 and p.N1156S (c.3467 A>A/G) at exon 22; the former is a newly identified mutation. Following diagnosis, treatment with miglustat was started based on a previously published guideline [5], and we allowed for some additional time to evaluate the effect of the treatment on the patient.

## 3. Discussion

The patient in this report is a typical case of juvenile onset form of NPC; however, the patient was initially misdiagnosed. Coexistence of his clumsiness and the bullying episode confounded our diagnosis; thus, his symptoms were misinterpreted as those associated with DCD and mediated by bullying. This course to diagnosis will provide instructive information for general pediatricians.

Difficulties in attention, short-term memory, and academic and social skills are more likely to be seen in children with DCD [4, 6]. In addition, being bullied is a common experience and may be related to emotional changes, even for normally developing children [7]. It is important to know that these mimicking conditions may arise as initial signs from large heterogeneous diseases in school-age children, such as adrenoleukodystrophy, subacute sclerosing panencephalitis, and NPC [8]. Psychiatric illnesses and other psychiatric signs, including attention deficit disorder, an Asperger-like presentation, and depressive disorder, have been emphasized as common presentations in adolescence or early adulthood patients [1, 9], but not so far in school-age children with NPC. The majority of NPC patients show characteristic vertical supranuclear gaze palsy, which is often overlooked at the early stage because slow pursuit is often maintained even if saccadic eye movements are already impaired [1]. The NPC Suspicion Index tool is useful for screening NPC based on heterogeneous presentations and family history [10], and it helps physicians unfamiliar with NPC in early identification of patients with suspicion of NPC. The coexistence of bullying may have delayed diagnosis in this case; however, whether or not there is bullying, careful documentation of patient history, physical examinations, and close follow-up are necessary when evaluating patients with multiple school problems.

Bone marrow examination should be considered when NPC is suspected, as the detection of cholesterol accumulating cells in the bone marrow is reported to be helpful for diagnosis before biochemical and histological detection in cultured skin fibroblasts [11]. Cytology of the patient's bone marrow made us strongly suspect NPC as an underlying cause, and the diagnosis was finally confirmed by cytochemical analysis of fibroblasts and genetic analysis.

Genotype-phenotype correlations of NPC are limited because most affected individuals are compound heterozygotes; however, several studies have shown some degree of prediction. No individuals with the I1061T NPC1 mutation, which is a frequent mutant allele in individuals of Western European descent, presented with the severe infantile onset form [12]. Mutations affecting the putative sterol-sensing domain, which is located between amino acid residues 615 and 797, can lead to the absence of stable NPC1 protein and severe neurological phenotype [13]. However, another survey in Japanese subjects did not reveal any clear phenotype-genotype relationships, although the numbers of patients were limited [14]. The patient in this report was found to carry the compound heterozygous mutations p.P836fsX838 [c.2506 (or 2507) del C] at exon 16 and p.N1156S (c.3467 A>A/G) at exon 22 in the* NPC1* gene. The former is a newly identified mutation that yields a stop codon downstream of the mutation and the latter missense mutation was previously reported as a pathogenic mutation [2]. Taken together, these mutations could be responsible for the NPC phenotype in this patient.

In the absence of any curative treatment, miglustat has been shown to delay the progression of the neurologic manifestations in a randomized clinical trial [15], and clinical practice settings. As an inhibitor of glycosphingolipids biosynthesis, it reduces lipid storage and cellular pathology in the brain, resulting in its therapeutic effects. It is currently considered as the only approved disease-specific therapy [5], and was approved for treatment of NPC in the EU in 2009 and Japan in 2012.

In summary, we have described a school-age child with juvenile onset form of NPC who developed difficulties in attention, short-term memory, and academic and social skills, and these problems were misinterpreted due to DCD and bullying. General pediatricians need to recognize that these multiple school problems could arise from neurodegenerative disorders and should consider NPC in differential diagnosis of children who present with progressive multiple school difficulties. We hope this report will lead to an improved approach for early diagnosis of children with onset of neurodegenerative disorders and allow better management of NPC patients and their families.
