# Corrigendum to “Niemann-Pick Disease Type C Presenting as a Developmental Coordination Disorder with Bullying by Peers in a School-Age Child”

**DOI:** 10.1155/2017/4120361

**Published:** 2017-09-20

**Authors:** Ryo Suzuki, Atsushi Tanaka, Toshiharu Matsui, Tetsuki Gunji, Jun Tohyama, Aya Narita, Eiji Nanba, Kousaku Ohno

**Affiliations:** ^1^Department of Pediatrics, Nagaoka Chuo General Hospital, 2041 Kawasaki-cho, Nagaoka, Niigata 940-8653, Japan; ^2^Department of Child Neurology, Nishi-Niigata Chuo National Hospital, 1-14-1 Masago, Nishi-ku, Niigata, Niigata 950-2085, Japan; ^3^Division of Child Neurology, Institute of Neurological Sciences, Faculty of Medicine, Tottori University, 36-1 Nishimachi, Yonago, Tottori 683-8504, Japan; ^4^Division of Functional Genomics, Research Center for Bioscience and Technology, Tottori University, 86 Nishimachi, Yonago, Tottori 683-8503, Japan; ^5^Sanin Rosai Hospital, 1-8-1 Kaike Shinden, Yonago, Tottori 683-8605, Japan

In the article titled “Niemann-Pick Disease Type C Presenting as a Developmental Coordination Disorder with Bullying by Peers in a School-Age Child” [1], the name of the Sixth author was given incorrectly as Aya Nairita. The author's name should have been written as Aya Narita. The revised authors' list is shown above.

## References

[1] Suzuki R., Tanaka A., Matsui T. (2015). Niemann-pick disease type C presenting as a developmental coordination disorder with bullying by peers in a school-age child. *Case Reports in Pediatrics*.

